# Gut–Brain Axis: Potential Factors Involved in the Pathogenesis of Parkinson's Disease

**DOI:** 10.3389/fneur.2020.00849

**Published:** 2020-08-25

**Authors:** Yin-Xia Chao, Muhammad Yaaseen Gulam, Nicholas Shyh Jenn Chia, Lei Feng, Olaf Rotzschke, Eng-King Tan

**Affiliations:** ^1^Department of Neurology, National Neuroscience Institute, Singapore, Singapore; ^2^Department of Neurology, Singapore General Hospital, Singapore, Singapore; ^3^Duke NUS Medical School, Singapore, Singapore; ^4^Department of Psychological Medicine, Yong Loo Lin School of Medicine, National University of Singapore, Singapore, Singapore; ^5^Singapore Immunology Network, Agency for Science, Technology and Research, Singapore, Singapore

**Keywords:** Parkinson's disease (PD), gut, genetics, microbiome, diet

## Abstract

Increasing evidence suggests an association between gastrointestinal (GI) disorders and susceptibility and progress of Parkinson's disease (PD). Gut–brain axis has been proposed to play important roles in the pathogenesis of PD, though the exact pathophysiologic mechanism has yet to be elucidated. Here, we discuss the common factors involved in both PD and GI disorders, including genes, altered gut microbiota, diet, environmental toxins, and altered mucosal immunity. Large-scale prospective clinical studies are needed to define the exact relationship between dietary factors, microbiome, and genetic factors in PD. Identification of early diagnostic markers and demonstration of the efficacy of diet modulation and regulation of gut microbiome through specific therapeutics can potentially change the treatment paradigm for PD.

## Introduction

Parkinson's disease (PD) is a common neurodegenerative disorder affecting 1–2 per 1,000 of the population (1). The incidence rate is generally lower for individuals before the age of 50 years, and it increases steadily with advanced age, peaking at 80 years old (2). The pathological hallmark in PD is the presence of intraneuronal aggregated alpha-synuclein (α-syn), Lewy body formation, and progressive loss of dopaminergic neurons in the substantia nigra compacta (SNc) which leads to the typical clinical symptoms including tremor, rigidity, bradykinesia, and posture instability (1). Current treatment for PD is largely symptomatic.

**Figure 1:**
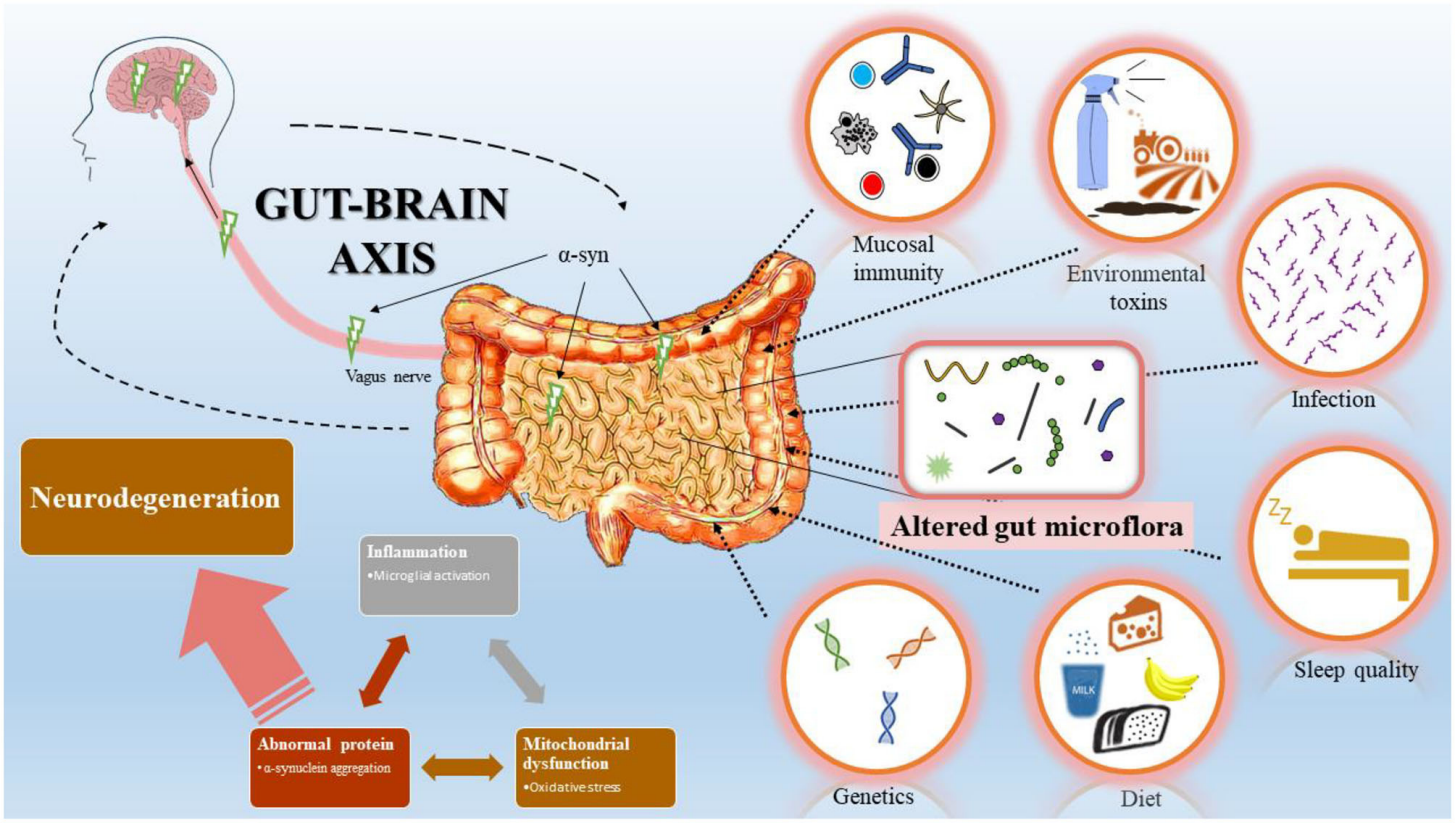
Bi-directional interaction between gastrointestinal (GI) tract and central nervous system (CNS). Schematic representation summarizes Braak's model of Parkinson's disease (PD) progression initiated from the GI tract. Changes in GI mucosal immunity, environmental toxins, infection, sleep quality, diet, and genetics modify the gut microflora and induce inflammation, mitochondrial dysfunction, and abnormal protein accumulation. Accumulation of α-syn in the GI tract spread via the vagus nerve to the CNS and leads to dopaminergic neuron degeneration.

Although motor symptoms are characteristic in PD, non-motor abnormalities in pre-PD phase are increasingly recognized. Among those, constipation is a prodromal marker in research diagnostic criteria for PD and may be an early manifestation of PD pathophysiology (3–5). The extent of the observed severity of the manifestation, especially the duration preceding PD, is unclear (3). However, several studies associate gastrointestinal (GI) dysfunction as a risk factor for PD development, with an early prevalence of 20% pre-PD diagnosis and 50% of the PD cases post-diagnosis (6, 7). Moreover, the association with GI dysfunction corroborates the well-established Braak's theory that PD initiation might begin in the GI tract, supported by the presence of Lewy body burden in the enteric nervous system (ENS) compared with other body regions and in the central nervous system (CNS) (8, 9). This has led to considerable interests to understand the etiology and presentation of pre-motor symptoms in PD patients. This review highlights the current findings linking pathophysiologic mechanisms between CNS and ENS in PD (Figure 1).

### Braak's Hypothesis and α-Synuclein

Emerging evidences have shown that PD involves not only the brain but also outside the CNS including the GI system (10, 11). Some propose the idea of a prion-like spread whereas others believe that it involves an interplay of multiple complex molecular mechanisms, including the well-known Braak's dual-hit model (12–15). According to Braak et al., the bi-directional communication between the network of neurons in the GI tract and the neurons of the CNS forms the gut–brain axis (10). Though criticisms argue that not all PD patients have the specific α-syn spreading pattern proposed by Braak, Braak's hypothesis suggests disease initiation and progression in a systematic manner in sporadic PD (14).

Braak et al. initial suggestion was an involvement of a neurotrophic agent or an unknown pathogenic insult in the GI tract (9). They went on to propose a six-stage system of PD progression in the brain and surrounding olfactory regions based on observed α-syn spreading patterns (16), and this can be linked to the many clinical features, and motor and non-motor syndromes of Parkinsonism (17, 18). Moreover, evidence of α-syn aggregations at olfactory bulbs (OBs), the ENS, and submucosal plexuses was associated with different pathologies observed in PD (17). Further studies underline the fact that the invading neurotrophic agent may either be a GI-initiated trigger by the intestinal microbiota or a toxin/pathogen from an external environment entering through the olfactory route (9, 19). As a consequence, this invasion promotes a pro-inflammatory intestinal mucosal environment, increases intestinal barrier permeability, which leads to the accumulation of reactive oxygen species (ROS), and creates an unbalanced homeostasis activating various immune mechanisms, which may ultimately trigger α-syn aggregation (14). It was increasingly evident that the initiation and spreading projected from two pathways, olfactory and GI tract (20, 21). Projecting neurons create a path via the vagal nerve and the dorsal motor nucleus of the vagus nerve (DMV) in the medulla (21). The aggregated α-syn was postulated to ascend anterogradely from the OB and retrogradely from the plexus of the GI tract via the vagus nerve (21). The α-syn aggregates propagate trans-synaptically to the DMV and eventually other regions of the CNS (15, 16, 21).

### Common Factors in the Pathogenesis of PD and Gastrointestinal Disorders

Here, we review potential factors involved in the association of GI disorders and PD, focusing on the common genetic factors, gut microbiota, and mucosal immunity. The environmental factors such as diet and environmental toxins together with potential role of sleep disorder will also be briefly discussed.

#### Genetic Factors

While most PD are sporadic with unknown etiologies, monogenic forms of PD and common genetic risk variants in sporadic PD have been identified (1, 22, 23). Carriers of pathogenic gene mutations frequently have indistinguishable clinical presentation from non-carriers (24).

Leucine-rich repeat kinase 2 (LRRK-2) is the most common genetic cause of autosomal dominant PD, accounting for 10–40% of familial cases in different populations (25). Genome-wide association studies (GWAS) show that some PD-associated LRRK2 variants are also independently associated with inflammatory bowel diseases (IBDs) (1, 23, 26, 27). More than 100 putative mutations have been reported in LRRK2 gene, though only six have been consistently shown to cause diseases, with two of these mutations G2019S and R1441C most commonly reported (28). Among the many functions of LRRK-2, the key roles include α-syn clearance and regulating the inflammatory response (22).

The genetic basis for IBD, in particular, Crohn's disease (CD) and ulcerative colitis (UC), has been supported by GWAS, which also suggested that some GWAS loci may also be associated with risk for PD (27, 29–31). This may be caused by susceptible individuals having an impaired mucosal immune response to GI commensals (29, 32). A Danish study made a similar association between PD and IBD in their cohort comprising IBD and non-IBD population (22). Apart from immune involvement, the authors also observed prominent differences in the gut microbiota in both CD and UC patients (22). These changes may have enabled the formations of Lewy pathology observed in PD, which can eventually through gut–brain neuronal interactions spread throughout the body (14).

#### Gut Microbiota

The involvement of gut microbiota in α-syn aggregation in PD has received increasing attention in the past several years (33, 34). Sampson and colleagues had shown that orally giving microbial metabolites can cause neuroinflammation in germ-free mice which leads to motor symptom development (35). Remarkably, microbiota transplants from PD patients exaggerated motor symptoms in α-syn-overexpressing mice compared with healthy controls. Other studies also suggested the synergistic role of gut microbiota in α-syn pathophysiology and neurodegeneration (36).

Gram-negative bacterium Helicobacter pylori causes gastritis and various GI problems, especially peptic ulcers (37–39). The association between PD and *H. pylori* was highlighted by Altschuler who noted the presence of duodenal ulcers in many clinical situations and suggested a probable causal link with idiopathic PD (40). Meta-analyses comparing healthy and *H. pylori*–affected individuals demonstrate a clear association between *H. pylori* and PD (39, 41). However, disease progression can be multifactorial, and it is impossible to single out a direct cause. Several investigators proposed various mechanisms of action associating *H. pylori* with PD pathogenesis. First, it is possible that *H. pylori* could be releasing CNS toxins vacuolating toxin, Vag A, and cytotoxin-associated gene, Cag A (37). Second, the damage can be through *H. pylori–*mediated glycosylation to generate cholesteryl glucosides, similar in form to toxin cycads (37, 42). These cholesteryl glucosides are neurotoxic, and they cross the blood–brain barrier (BBB) to cause dopaminergic neuron degeneration (37). Third, *H. pylori* can activate immune mechanisms, monocytes, eicosanoids, interleukins, and cytokines (TNF-α, IL-10, IL-6, IL-8, IL-1B, IL-13), resulting in an exaggerated neuroinflammatory response, leading to disruption and infiltration in the BBB, microgliosis, and neurodegeneration (39). Fourth, *H. pylori* can initiate apoptosis through apoptotic pathways such as the nitric oxide and mitochondrial Fas–FasL pathway, causing neurodegeneration (39). Lastly, the production of autoantibodies against dopaminergic neurons induced by *H. pylori* and host antigens can lead to widespread neuroinflammation (37, 39).

More recently, Wallen et al. conducted an association study (MWAS) between microbiome and PD using two large datasets. They found that the opportunistic pathogens and carbohydrate-metabolizing probiotics were significantly increased while short-chain fatty acid (SCFA)–producing bacteria were decreased in PD patients (43). These findings will facilitate testing the potential role of some of these pathogens in PD pathogenesis.

#### Diet

The association between diet, nutritional status, and PD pathogenesis has also attracted considerable attention after studies on the existence of the gut–brain axis and gut microbiota (22). Reduction in gut commensal Prevotellaceae composition reduces mucin synthesis increasing gut leakiness, affecting the production of SCFA involved in thiamine and folate biosynthesis, and the increase in Lactobacilliceae can alter gut hormone ghrelin which can modify nigrostriatal dopamine neuronal integrity (19). SCFAs can also exert a systemic anti-inflammatory response increasing ROS, which can lead to synucleinopathy (14, 19).

Moreover, celiac disease, a gluten-induced gastrointestinal disorder, has been reported to be associated with PD pathogenesis. Based on the results from a pilot study, 2 out of 67 celiac disease patients from the cohort reported PD symptoms (44). When these patients underwent a diet alteration to a more gluten-free one, their symptoms improved (45). Although these studies are preliminary, further investigation should be conducted with a larger cohort to illustrate this association and the importance of diet in PD.

#### Mucosal Immunity

The intestinal lumen encompasses the most extensive enviro-host interface, continuously interrogated by a high antigenic load resulting from exposures to deadly pathogens, diet changes, and commensals (32). Existing immune systems and co-evolving microbial community are reciprocal, and there are mandatory checkpoints available to ensure an appropriate response to a pathogenic insult (46). These systems continue to regulate and shape its response, accommodating to the changes observed throughout the host's lifetime (46).

The cellular aspects of GALT and the epithelial barrier comprise the localized microenvironment, lymphoid follicles, mesenteric lymph nodes, and Peyer's and colonic patches, whereas the molecular compartment consists of T and B regulatory cells, intraepithelial lymphocytes (IELs), innate lymphoid cells, macrophages, and dendritic cells (46, 47). GALT, especially the immune cells in the appendix, were recently found unique for PD pathogenesis (48). The epithelial barrier and the cells of the intestinal epithelium are the first lines of defense against any invading pathogen (32, 46). Its unique structure functions to provide a physical barrier, drawing a forefront rich with antimicrobial peptides, immunoglobulins A (IgA), and a tight monolayer preventing bacterial penetrations (32, 46). Although there were contradicting observations on the noticeable structural changes in a disease state, many agree that the most imminent damage occurs to the tight monolayer (49). Epithelial dysfunction demonstrated in 1-methyl-4-phenyl-1,2,3,6-tetrahydropiridine (MPTP) animal models demonstrated noticeable differences in expression patterns of ZO-1, occludin, and tight-junction proteins (32, 50). Indeed, colonic biopsies from PD individuals confirm this observation (50).

Regulatory cells (Tregs) are a subset of CD4^+^ T cells that hamper the progression of IBDs and provide peripheral tolerance (32). Among the many functions of Tregs, one which is worth mentioning is its ability to act as a negative regulator, aimed at curtailing a pro-inflammatory situation presented by effector (Teff) cells (32). They achieve this by actively secreting cytokines (IL-10, TGF-β) and cytotoxic T-lymphocyte antigen, CTLA 4 (32).

It would be apt to describe the characteristic features of IBD as a disease with a defective T-cell signaling, mostly imbalances between Treg and Th17, along with an altered cytokine profile (32, 51). Both Th17 and Treg cells originate from a common CD4^+^ precursor cell, mediated by TGF-β signal (52). However, their fates differ at the end stage of differentiation (52). As opposed to Treg's function of maintaining intestinal homeostasis, Th17 cells initiate gut inflammation (51). In addition, commensal microbiota and bacterial metabolites can also positively or negatively alter cytokine profiles, inducing the pathway toward Treg or Th1/Th17 formations (32, 52). Supporting this observation, independent findings on PD patients' colonic biopsies and inflammatory diseases both indicate an exaggerated inflammation with extreme amounts of pro-inflammatory (TNF, IL-1β, IFNγ, IL-5) molecules (22, 32). Co-culture of autologous Th17 cells and stem cell–derived dopaminergic (DA) neurons showed that Th17 cells can kill the DA neurons through releasing of IL-17A (35). Whether these DA neuron–specific Th17 cells are from the mucosal immunity is unknown.

There are other relevant cells of the immune system with a primary role to function constitutively with other immune cells to maintain homeostasis in PD. They provide a supportive role in ensuring inflammation control and immune surveillance. For instance, the intestinal epithelial cells (IECs) of the epithelium secrete IgA, antimicrobial proteins, and anti-inflammatory cytokines with crucial roles in differentiation, maturation, migration, and response (32). Similarly, another cell population found alongside IECs are the IELs (32, 47). IELs are T cells with a T-cell receptor which have come in contact with antigens and have differentiated in either natural IEL or induced IELs (32). Although they take on separate differentiation patterns, their central role is to maintain intestinal homeostasis (32). They secrete pro-inflammatory (IFNγ and TNF) cytokines, provide immune surveillance through migration to intestinal epithelial surface, which is in close contact with pathogens, and produce IL-10 and TGF-β suppressing intestinal inflammation (32). Likewise, regulatory B cells (Bregs), antibody-producing cells, which release cytokines (IL-10) are also involved in maintaining homeostasis and suppressing inflammation, and regulating the balance of Tregs, Th1, and Th17 (32).

The distinctive pattern of GI inflammation, especially at the early stages of the disease, with its signature symptoms, suggests the extent of the involvement of the mucosal immune system. It is unclear if α-syn aggregates were the cause or effect in the pathophysiology (53). Stolzenberg et al. concluded that α-syn secreted from enteric nerves of a pro-inflammatory ENS is the cause of GI inflammation, and it also acts as a chemoattractant for neutrophils and monocytes perpetuating the condition (53).

#### Environmental Toxins

The link between herbicide and paraquat exposure and neurotoxin MPTP administration and PD has suggested that environmental toxins can cause the disease. A recent meta-analysis from 31 studies with occupational exposure to pesticides suggested a significant association with PD risk (54). Rotenone has been reported to inhibit mitochondrial complex 1 activity, whereas paraquat causes oxidative stress (55–58). The gram-negative bacteria endotoxin lipopolysaccharides (LPS) have also been reported to induce dopaminergic neuron death in animal models (59–61). Supporting Braak's theory of a peripheral-to-central spread, agrochemicals such as metals, pesticides, and herbicides that enter the body via inhalation and/or ingestion are suggested to be a possible initiator causing widespread inflammation and mitochondrial dysfunction which ultimately lead to abnormal α-syn accumulation and dopaminergic neuron degeneration in the midbrain (10, 59, 62). Moreover, an established causal link between agrochemical use and PD can be challenging as the time between exposure and symptom presentation has a long latency period (10 to 20 years) (62). Hence, epidemiological studies have to improve their assessment methodologies, employ neurologists for diagnostics, and redefine the way they study past exposures accurately (63).

#### Sleep Quality

Sleep disorder is one of the non-motor symptoms reported in PD patients in the prodromal phase (64, 65). Interestingly, sleep disturbance has also been reported in IBD patients (66, 67). The underlying mechanisms for the sleep disturbance in PD and IBD are yet to be elucidated.

## Conclusions and Future Perspectives

The etiology of PD involves both genetic and environmental factors. The gut is one of the major systems exposed to the environment directly and connects to the brain. Understanding the gut–brain axis has allowed us to appreciate the development and progression of the disease considerably. The GI system (which consists of the microbiome) is continuously being influenced by various factors, such as environment, diet, infection, and mucosal immunity. The overlapping genetic factors between PD and GI disorders suggest common etiologic links between the GI system and PD development. Given that the current treatments for PD are mainly symptomatic, regulation of the gut microbiota and mucosal immunity through diet, such as giving probiotics, may have protective effect in PD treatment. The association of PD with GI system may provide prophylactic and targeted PD therapy in selected risk individuals.

Large-scale prospective clinical studies are needed to define the exact relationship between dietary factors, microbiome, and genetic factors in PD. Identification of early diagnostic markers and demonstration of the efficacy of diet modulation and regulation of gut microbiome through specific therapeutics can potentially change the treatment paradigm for PD.

## Author Contributions

Y-XC and E-KT planned the outline of the manuscript. MG prepared the draft. NC, Y-XC, E-KT, LF, and OR revised the manuscript. All authors contributed to the article and approved the submitted version.

## Conflict of Interest

The authors declare that the research was conducted in the absence of any commercial or financial relationships that could be construed as a potential conflict of interest.

## References

[1] Tysnes OB Storstein A. Epidemiology of Parkinson's disease. J Neural Transm. (2017) 124:901–5. 10.1007/s00702-017-1686-y 28150045

[2] Ascherio A Schwarzschild MA. The epidemiology of Parkinson's disease: risk factors and prevention. Lancet Neurol. (2016) 15:1257–72. 10.1016/S1474-4422(16)30230-7 27751556

[3] Kujawska M Jodynis-Liebert J. What is the evidence that Parkinson's disease is a prion disorder, which originates in the gut? Int J Mol Sci. (2018) 19:3573. 10.3390/ijms19113573 30424585 PMC6274907

[4] Berg D Postuma RB Adler CH Bloem BR Chan P Dubois B MDS research criteria for prodromal Parkinson's disease. Mov Disord. (2015) 30:1600–11. 10.1002/mds.26431 26474317

[5] Heinzel S Berg D Gasser T Chen H Yao C Postuma RB. Update of the MDS research criteria for prodromal Parkinson's disease. Mov Disord. (2019) 34:1464–70. 10.1002/mds.27802 31412427

[6] Adams-Carr KL Bestwick JP Shribman S Lees A Schrag A Noyce AJ. Constipation preceding Parkinson's disease: a systematic review and meta-analysis. J Neurol Neurosurg Psychiatry. (2016) 87:710–6. 10.1136/jnnp-2015-311680 26345189

[7] Knudsen K Krogh K Ostergaard K Borghammer P. Constipation in Parkinson's disease: subjective symptoms, objective markers, and new perspectives. Mov Disord. (2017) 32:94–105. 10.1002/mds.26866 27873359

[8] Lin CH Lin JW Liu YC Chang CH Wu RM. Risk of Parkinson's disease following severe constipation: a nationwide population-based cohort study. Parkinsonism Relat Disord. (2014) 20:1371–5. 10.1016/j.parkreldis.2014.09.026 25293395

[9] Braak H Rub U Gai WP Del Tredici K. Idiopathic Parkinson's disease: possible routes by which vulnerable neuronal types may be subject to neuroinvasion by an unknown pathogen. J Neural Transm. (2003) 110:517–36. 10.1007/s00702-002-0808-2 12721813

[10] Klingelhoefer L Reichmann H. Pathogenesis of Parkinson disease–the gut-brain axis and environmental factors. Nat Rev Neurol. (2015) 11:625–36. 10.1038/nrneurol.2015.197 26503923

[11] Lionnet A Leclair-Visonneau L Neunlist M Murayama S Takao M Adler CH. Does Parkinson's disease start in the gut? Acta Neuropathol. (2018) 135:1–12. 10.1007/s00401-017-1777-8 29039141

[12] Brundin P Melki R. Prying into the prion hypothesis for Parkinson's disease. J Neurosci. (2017) 37:9808–18. 10.1523/JNEUROSCI.1788-16.2017 29021298 PMC5637113

[13] Surmeier DJ Obeso JA Halliday GM. Parkinson's disease is not simply a prion disorder. J Neurosci. (2017) 37:9799–807. 10.1523/JNEUROSCI.1787-16.2017 29021297 PMC5637112

[14] Rietdijk CD Perez-Pardo P Garssen J van Wezel RJ Kraneveld AD. Exploring Braak's hypothesis of Parkinson's disease. Front Neurol. (2017) 8:37. 10.3389/fneur.2017.00037 28243222 PMC5304413

[15] Visanji NP Brooks PL Hazrati LN Lang AE. The prion hypothesis in Parkinson's disease: braak to the future. Acta Neuropathol Commun. (2013) 1:2. 10.1186/2051-5960-1-2 24252164 PMC3776210

[16] Braak H Del Tredici K Rub U de Vos RA Jansen Steur EN Braak E. Staging of brain pathology related to sporadic Parkinson's disease. Neurobiol Aging. (2003) 24:197–211. 10.1016/S0197-4580(02)00065-9 12498954

[17] Braak H de Vos RA Bohl J Del Tredici K. Gastric alpha-synuclein immunoreactive inclusions in Meissner's and Auerbach's plexuses in cases staged for Parkinson's disease-related brain pathology. Neurosci Lett. (2006) 396:67–72. 10.1016/j.neulet.2005.11.012 16330147

[18] Shannon KM Keshavarzian A Mutlu E Dodiya HB Daian D Jaglin JA. Alpha-synuclein in colonic submucosa in early untreated Parkinson's disease. Mov Disord. (2012) 27:709–15. 10.1002/mds.23838 21766334

[19] Perez-Pardo P Kliest T Dodiya HB Broersen LM Garssen J Keshavarzian A. The gut-brain axis in Parkinson's disease: possibilities for food-based therapies. Eur J Pharmacol. (2017) 817:86–95. 10.1016/j.ejphar.2017.05.042 28549787

[20] Hawkes CH Del Tredici K Braak H. Parkinson's disease: a dual-hit hypothesis. Neuropathol Appl Neurobiol. (2007) 33:599–614. 10.1111/j.1365-2990.2007.00874.x 17961138 PMC7194308

[21] Hawkes CH Del Tredici K Braak H. Parkinson's disease: the dual hit theory revisited. Ann N Y Acad Sci. (2009) 1170:615–22. 10.1111/j.1749-6632.2009.04365.x 19686202

[22] Villumsen M Aznar S Pakkenberg B Jess T Brudek T. Inflammatory bowel disease increases the risk of Parkinson's disease: a Danish nationwide cohort study 1977-2014. Gut. (2019) 68:18–24. 10.1136/gutjnl-2017-315666 29785965

[23] Ylonen S Siitonen A Nalls MA Ylikotila P Autere J Eerola-Rautio J. Genetic risk factors in Finnish patients with Parkinson's disease. Parkinsonism Relat Disord. (2017) 45:39–43. 10.1016/j.parkreldis.2017.09.021 29029963 PMC5812481

[24] Healy DG Falchi M O'Sullivan SS Bonifati V Durr A Bressman S. Phenotype, genotype, and worldwide genetic penetrance of LRRK2-associated Parkinson's disease: a case-control study. Lancet Neurol. (2008) 7:583–90. 10.1016/S1474-4422(08)70117-0 18539534 PMC2832754

[25] Hardy J. Genetic analysis of pathways to Parkinson disease. Neuron. (2010) 68:201–6. 10.1016/j.neuron.2010.10.014 20955928 PMC2997424

[26] Liu Z Lenardo MJ. The role of LRRK2 in inflammatory bowel disease. Cell Res. (2012) 22:1092–4. 10.1038/cr.2012.42 22430149 PMC3391018

[27] Witoelar A Jansen IE Wang Y Desikan RS Gibbs JR Blauwendraat C. Genome-wide pleiotropy between Parkinson disease and autoimmune diseases. JAMA Neurol. (2017) 74:780–92. 10.1001/jamaneurol.2017.0469 28586827 PMC5710535

[28] Kluss JH Mamais A Cookson MR. LRRK2 links genetic and sporadic Parkinson's disease. Biochem Soc Trans. (2019) 47:651–61. 10.1042/BST20180462 30837320 PMC6563926

[29] Franke A McGovern DP Barrett JC Wang K Radford-Smith GL Ahmad T. Genome-wide meta-analysis increases to 71 the number of confirmed Crohn's disease susceptibility loci. Nat Genet. (2010) 42:1118–25. 10.1038/ng.717 21102463 PMC3299551

[30] Anderson CA Boucher G Lees CW Franke A D'Amato M Taylor KD. Meta-analysis identifies 29 additional ulcerative colitis risk loci, increasing the number of confirmed associations to 47. Nat Genet. (2011) 43:246–52. 10.1038/ng.764 21297633 PMC3084597

[31] McGovern DP Gardet A Torkvist L Goyette P Essers J Taylor KD. Genome-wide association identifies multiple ulcerative colitis susceptibility loci. Nat Genet. (2010) 42:332–7. 10.1038/ng.549 20228799 PMC3087600

[32] Sun M He C Cong Y Liu Z. Regulatory immune cells in regulation of intestinal inflammatory response to microbiota. Mucosal Immunol. (2015) 8:969–78. 10.1038/mi.2015.49 26080708 PMC4540654

[33] Scheperjans F Aho V Pereira PA Koskinen K Paulin L Pekkonen E. Gut microbiota are related to Parkinson's disease and clinical phenotype. Mov Disord. (2015) 30:350–8. 10.1002/mds.26069 25476529

[34] Yang D Zhao D Ali Shah SZ Wu W Lai M Zhang X The role of the gut microbiota in the pathogenesis of Parkinson's disease. Front Neurol. (2019) 10:1155 10.3389/fneur.2019.01155 31781020 PMC6851172

[35] Sampson TR Debelius JW Thron T Janssen S Shastri GG Ilhan ZE. Gut microbiota regulate motor deficits and neuroinflammation in a model of Parkinson's disease. Cell. (2016) 167:1469–80 e12. 10.1016/j.cell.2016.11.018 27912057 PMC5718049

[36] Gorecki AM Preskey L Bakeberg MC Kenna JE Gildenhuys C MacDougall G. Altered gut microbiome in parkinson's disease and the influence of lipopolysaccharide in a human alpha-synuclein over-expressing mouse model. Front Neurosci. (2019) 13:839. 10.3389/fnins.2019.00839 31440136 PMC6693556

[37] McGee DJ Lu XH Disbrow EA. Stomaching the possibility of a pathogenic role for *Helicobacter pylori* in Parkinson's disease. J Parkinsons Dis. (2018) 8:367–74. 10.3233/JPD-181327 29966206 PMC6130334

[38] Mridula KR Borgohain R Chandrasekhar Reddy V Bandaru V Suryaprabha T. Association of *Helicobacter pylori* with Parkinson's disease. J Clin Neurol. (2017) 13:181–6. 10.3988/jcn.2017.13.2.181 28406585 PMC5392461

[39] Dardiotis E Tsouris Z Mentis AA Siokas V Michalopoulou A Sokratous M. *H. pylori* and Parkinson's disease: meta-analyses including clinical severity. Clin Neurol Neurosurg. (2018) 175:16–24. 10.1016/j.clineuro.2018.09.039 30308319

[40] Altschuler E. Gastric *Helicobacter pylori* infection as a cause of idiopathic Parkinson disease and non-arteric anterior optic ischemic neuropathy. Med Hypotheses. (1996) 47:413–4. 10.1016/S0306-9877(96)90223-6 8951807

[41] Shen X Yang H Wu Y Zhang D Jiang H. Meta-analysis: association of *Helicobacter pylori* infection with Parkinson's diseases. Helicobacter. (2017) 22. 10.1111/hel.12398 28598012

[42] Schulz JD Hawkes EL Shaw CA. Cycad toxins, *Helicobacter pylori* and parkinsonism: cholesterol glucosides as the common denomenator. Med Hypotheses. (2006) 66:1222–6. 10.1016/j.mehy.2004.12.033 16488551

[43] Wallen ZD Appah M Dean MN Sesler CL Factor SA Molho E. Characterizing dysbiosis of gut microbiome in PD: evidence for overabundance of opportunistic pathogens. NPJ Parkinsons Dis. (2020) 6:11. 10.1038/s41531-020-0112-6 32566740 PMC7293233

[44] Burk K Farecki ML Lamprecht G Roth G Decker P Weller M. Neurological symptoms in patients with biopsy proven celiac disease. Mov Disord. (2009) 24:2358–62. 10.1002/mds.22821 19845007

[45] Di Lazzaro V Capone F Cammarota G Di Giuda D Ranieri F. Dramatic improvement of Parkinsonian symptoms after gluten-free diet introduction in a patient with silent celiac disease. J Neurol. (2014) 261:443–5. 10.1007/s00415-014-7245-7 24464413

[46] Garrett WS Gordon JI Glimcher LH. Homeostasis and inflammation in the intestine. Cell. (2010) 140:859–70. 10.1016/j.cell.2010.01.023 20303876 PMC2845719

[47] Wershil BK Furuta GT. 4. Gastrointestinal mucosal immunity. J Allergy Clin Immunol. (2008). 121(Suppl. 2):S380–3. 10.1016/j.jaci.2007.10.023 18241686

[48] Killinger B Labrie V. The appendix in Parkinson's disease: from vestigial remnant to vital organ? J Parkinsons Dis. (2019) 9:S345–58. 10.3233/JPD-191703 31609697 PMC6839473

[49] Clairembault T Leclair-Visonneau L Coron E Bourreille A Le Dily S Vavasseur F. Structural alterations of the intestinal epithelial barrier in Parkinson's disease. Acta Neuropathol Commun. (2015) 3:12. 10.1186/s40478-015-0196-0 25775153 PMC4353469

[50] Fang X. Impaired tissue barriers as potential therapeutic targets for Parkinson's disease and amyotrophic lateral sclerosis. Metab Brain Dis. (2018) 33:1031–43. 10.1007/s11011-018-0239-x 29681010

[51] Vidal PM Pacheco R. Targeting the dopaminergic system in autoimmunity. J Neuroimmune Pharmacol. (2019) 15:57–73. 10.1007/s11481-019-09834-5 30661214

[52] Lee GR. The balance of Th17 versus treg cells in autoimmunity. Int J Mol Sci. (2018) 19:730. 10.3390/ijms19030730 29510522 PMC5877591

[53] Stolzenberg E Berry D Yang Lee EY Kroemer A Kaufman S. A role for neuronal alpha-synuclein in gastrointestinal immunity. J Innate Immun. (2017) 9:456–63. 10.1159/000477990 28651250 PMC5865636

[54] Gunnarsson LG Bodin L. Occupational exposures and neurodegenerative diseases-a systematic literature review and meta-analyses. Int J Environ Res Public Health. (2019) 16:337. 10.3390/ijerph16030337 30691095 PMC6388365

[55] Petrovitch H Ross GW Abbott RD Sanderson WT Sharp DS Tanner CM. Plantation work and risk of Parkinson disease in a population-based longitudinal study. Arch Neurol. (2002) 59:1787–92. 10.1001/archneur.59.11.1787 12433267

[56] Gao HM Hong JS Zhang W Liu B. Synergistic dopaminergic neurotoxicity of the pesticide rotenone and inflammogen lipopolysaccharide: relevance to the etiology of Parkinson's disease. J Neurosci. (2003) 23:1228–36. 10.1523/JNEUROSCI.23-04-01228.2003 12598611 PMC6742266

[57] Tanner CM Kamel F Ross GW Hoppin JA Goldman SM Korell M. Rotenone, paraquat, and Parkinson's disease. Environ Health Perspect. (2011) 119:866–72. 10.1289/ehp.1002839 21269927 PMC3114824

[58] Pan-Montojo F Schwarz M Winkler C Arnhold M O'Sullivan GA Pal A. Environmental toxins trigger PD-like progression via increased alpha-synuclein release from enteric neurons in mice. Sci Rep. (2012) 2:898. 10.1038/srep00898 23205266 PMC3510466

[59] Purisai MG McCormack AL Cumine S Li J Isla MZ Di Monte DA. Microglial activation as a priming event leading to paraquat-induced dopaminergic cell degeneration. Neurobiol Dis. (2007) 25:392–400. 10.1016/j.nbd.2006.10.008 17166727 PMC2001246

[60] Cai Z Fan LW Kaizaki A Tien LT Ma T Pang Y. Neonatal systemic exposure to lipopolysaccharide enhances susceptibility of nigrostriatal dopaminergic neurons to rotenone neurotoxicity in later life. Dev Neurosci. (2013) 35:155–71. 10.1159/000346156 23446007 PMC3777222

[61] Brudek T. Inflammatory Bowel diseases and Parkinson's disease. J Parkinsons Dis. (2019) 9:S331–44. 10.3233/JPD-191729 31609699 PMC6839501

[62] Ascherio A Chen H Weisskopf MG O'Reilly E McCullough ML Calle EE. Pesticide exposure and risk for Parkinson's disease. Ann Neurol. (2006) 60:197–203. 10.1002/ana.20904 16802290

[63] Breckenridge CB Berry C Chang ET Sielken RL Jr Mandel JS. Association between Parkinson's disease and cigarette smoking, rural living, well-water consumption, farming and pesticide use: systematic review and meta-analysis. PloS ONE. (2016) 11:e0151841. 10.1371/journal.pone.0151841 27055126 PMC4824443

[64] Aguirre-Mardones C Iranzo A Vilas D Serradell M Gaig C Santamaria J. Prevalence and timeline of nonmotor symptoms in idiopathic rapid eye movement sleep behavior disorder. J Neurol. (2015) 262:1568–78. 10.1007/s00415-015-7742-3 25929658

[65] Nihei Y Takahashi K Koto A Mihara B Morita Y Isozumi K. REM sleep behavior disorder in Japanese patients with Parkinson's disease: a multicenter study using the REM sleep behavior disorder screening questionnaire. J Neurol. (2012) 259:1606–12. 10.1007/s00415-011-6386-1 22231870

[66] Bazin T Micoulaud Franchi JA Terras N Taillard J Laharie D Zerbib F. Altered sleep quality is associated with Crohn's disease activity: an actimetry study. Sleep Breath. (2019) 10.1007/s11325-019-01934-z 31512092

[67] Sofia MA Lipowska AM Zmeter N Perez E Kavitt R Rubin DT Poor sleep quality in Crohn's disease is associated with disease activity and risk for hospitalization or surgery. Inflamm Bowel Dis. (2019) 26:1251–9. 10.1093/ibd/izz258 PMC7365809 31820780

