# Corrigendum: Gut–Brain Axis: Potential Factors Involved in the Pathogenesis of Parkinson's Disease

**DOI:** 10.3389/fneur.2020.625446

**Published:** 2020-11-27

**Authors:** Yin-Xia Chao, Muhammad Yaaseen Gulam, Nicholas Shyh Jenn Chia, Lei Feng, Olaf Rotzschke, Eng-King Tan

**Affiliations:** ^1^Department of Neurology, National Neuroscience Institute, Singapore, Singapore; ^2^Department of Neurology, Singapore General Hospital, Singapore, Singapore; ^3^Duke NUS Medical School, Singapore, Singapore; ^4^Department of Psychological Medicine, Yong Loo Lin School of Medicine, National University of Singapore, Singapore, Singapore; ^5^Singapore Immunology Network, Agency for Science, Technology and Research, Singapore, Singapore

**Keywords:** Parkinson's disease (PD), gut, genetics, microbiome, diet

In the original article, there was an error. The findings cited in Reference Number 43 (Wallen et al., 2020) were inaccurately stated.

A correction has been made to *Common factors in the Pathogenesis of PD and Gastrointestinal Disorders, Gut Microbiota, Paragraph 3*. The corrected paragraph is shown below:

More recently, Wallen et al. conducted an association study (MWAS) between microbiome and PD using two large datasets. They found that the opportunistic pathogens and carbohydrate-metabolizing probiotics were significantly increased while short-chain fatty acid (SCFA)–producing bacteria were decreased in PD patients (43). These findings will facilitate testing the potential role of some of these pathogens in PD pathogenesis.

The authors apologize for this error and state that this does not change the scientific conclusions of the article in any way. The original article has been updated.
